# A Rare Case of a Non‐Invasive Vaginal Urothelial Carcinoma Demonstrating a Clonal Relationship With a Metachronous Non‐Invasive Bladder Urothelial Carcinoma

**DOI:** 10.1155/crip/4360431

**Published:** 2026-09-17

**Authors:** Cassandra Hill, Noman Ghazanfar, Andy Chuang, Robert Gordon Hislop, Ketan Rathod, Jamie Wilson, Ghulam Mustafa Nandwani

**Affiliations:** ^1^ Ninewells Hospital and Medical School, NHS Tayside, Dundee, UK, dundee.ac.uk; ^2^ University of Dundee, Dundee, UK, dundee.ac.uk

**Keywords:** anterior pelvic exenteration, bladder urothelial carcinoma, clonality, vaginal urothelial carcinoma

## Abstract

Non‐invasive vaginal urothelial carcinomas (UCs) are extremely rare and are almost always associated with a non‐invasive bladder UC. The pathogenesis of these rare vaginal lesions is uncertain. They may represent a primary vaginal tumour or they may arise from a urinary tract UC, in which tumour cells “seed” by non‐invasive mechanisms. We describe a case of a non‐invasive vaginal UC, occurring 6 years following treatment of a non‐invasive bladder UC, managed with an anterior pelvic exenteration and followed up over 5 years. Molecular analysis using a genome‐wide single nucleotide polymorphism (SNP) microarray to assess copy number variations and regions of copy number neutral loss of heterozygosity (CN‐LOH) was performed on both tumours. These results evidence a clonal relationship between these two tumours indicating that the vaginal tumour is the result of non‐invasive tumour “seeding” from the original bladder lesion. To our knowledge this is the first case report to demonstrate clonality between metachronous non‐invasive bladder and vaginal UCs.

## 1. Background

Vaginal urothelial carcinomas (UCs) are rare, usually secondary to direct invasion, metastatic spread or pagetoid spread from a primary urinary tract UC [1–3]. Very occasionally, vaginal UCs are found without an associated invasive urinary tract UC with no evidence of a spatial connection [4–15]. No definite consensus exists as to the origin of these vaginal UCs; however, the majority of these cases are associated with a non‐invasive bladder UC [4–11, 13–15].

Non‐invasive bladder UCs are frequently multifocal and it is thought that vaginal UCs may represent an extraurinary tract manifestation of this process. Two hypotheses have been proposed to establish the pathogenesis of this multifocality: the monoclonality hypothesis and the field change hypothesis. The monoclonality hypothesis suggests that multifocal tumours arise via tumour cells superficially “seeding” from an original tumour. Whereas the field change hypothesis suggests that multiple separate polyclonal tumours arise within an area of carcinogenically transformed cells. Regarding bladder UCs, both of these concepts have supporting evidence within the literature [16] and may coexist. Whether vaginal UCs represent a de novo primary tumour or are secondary to a bladder UC which has spread by superficial mechanisms remains to be established.

Here, we describe a case of a non‐invasive vaginal UC occurring metachronously 6 years following a non‐invasive bladder UC. To investigate the pathogenesis of this rare lesion, we performed a whole genome single nucleotide polymorphism (SNP) microarray to determine if the two tumours were clonal.

## 2. Case Report

A 65‐year old woman, who presented with anaemia and weight loss, was referred to urology after an incidental discovery of a 5.2 cm × 5.7 cm bladder mass on a CT chest abdomen‐pelvis scan (CT CAP). She had a background of Crohn′s disease and hypertension. Her Eastern Cooperative Oncology Group performance status was zero. Cystoscopy revealed a large papillary lesion located on the left lateral bladder wall. A complete transurethral resection of bladder tumour (TURBT) was performed without evidence of bladder perforation. The resected tumour weighed 92 g. The histopathology showed a non‐invasive (pTa), high grade, G2 bladder UC with no evidence of CIS. Six weeks later, a re‐resection TURBT and quadrant bladder biopsies showed no residual tumour. Because of an international supply shortage, intravesical Bacillus Calmette–Guerin (BCG) was not given after discussion with the patient. The patient had cystoscopic and upper tract imaging surveillance, which showed no evidence of recurrence for 6 years.

**Figure 1:**
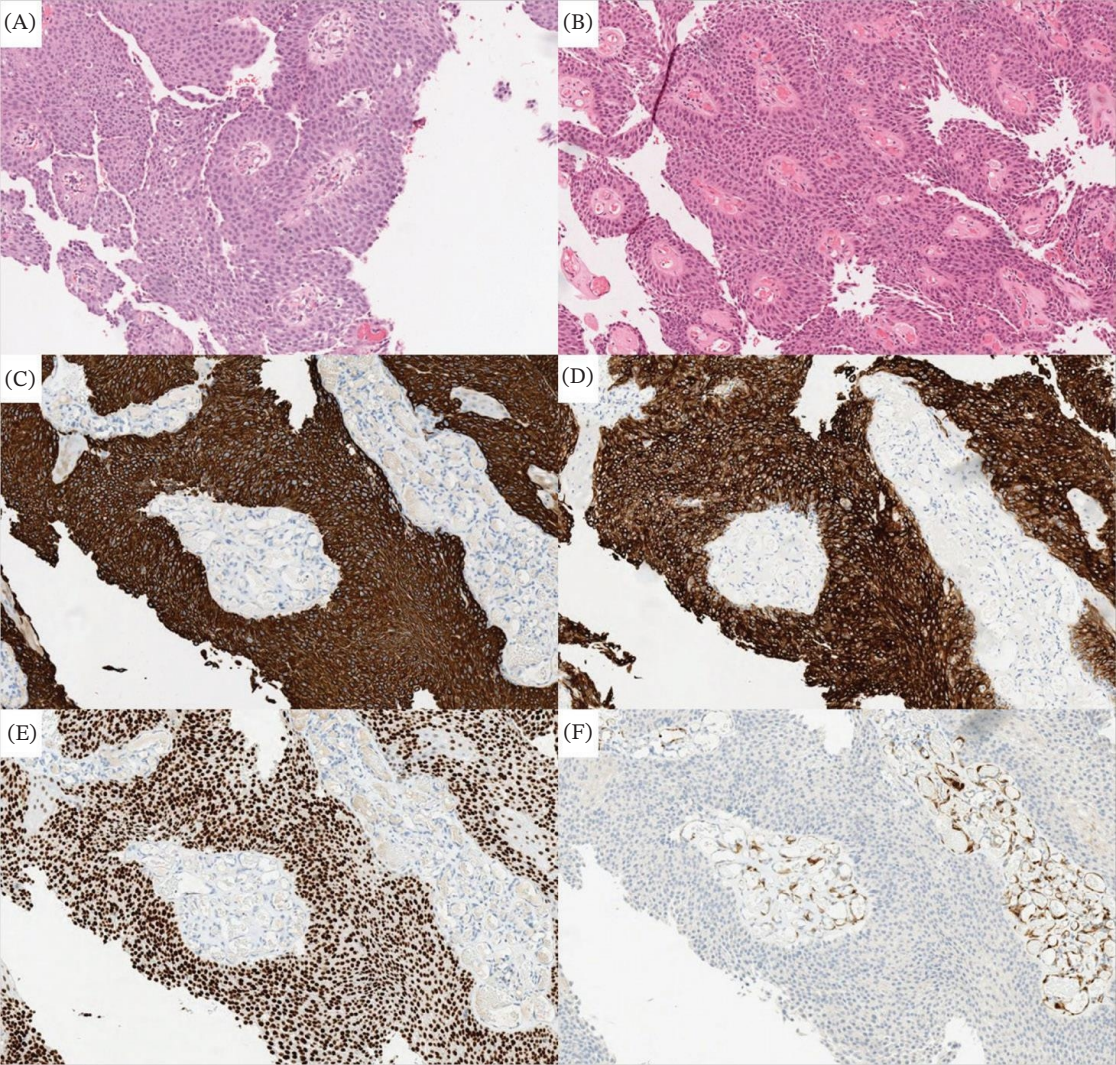
(A) The morphology of the initial bladder biopsy was comparable to the (B) morphology of the vaginal biopsy demonstrating a papillary architecture. Immunohistochemistry was performed on the vaginal biopsy. (C) CK7 and (D) CK20 both showed strong membranous and cytoplasmic staining. (E) GATA3 demonstrated strong nuclear positivity, while (F) p16 was negative.

**Figure 2:**
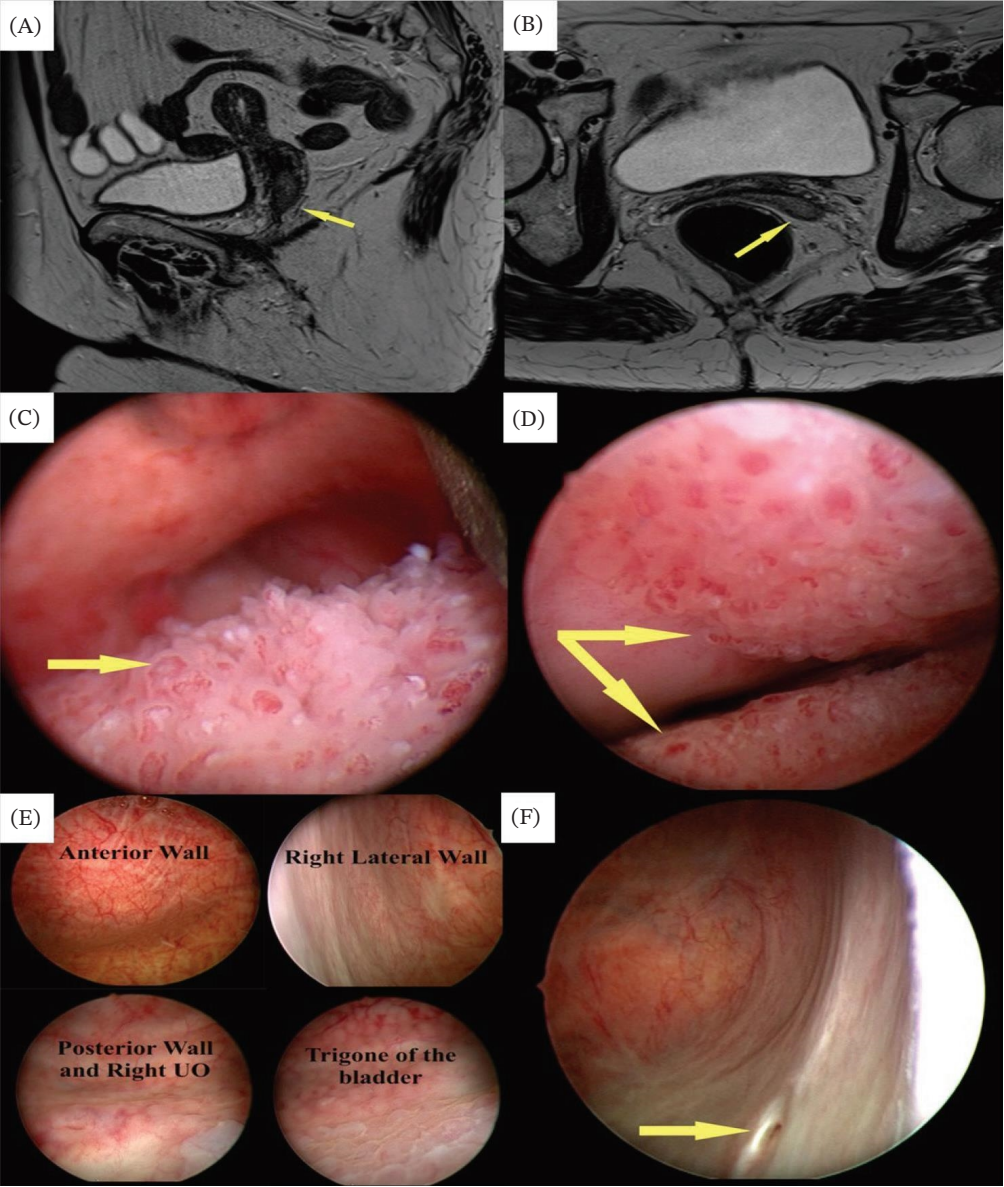
MRI scan showed asymmetrical thickening of the left side of the vagina (yellow arrows) consistent with a vaginal lesion and abnormal tissue in the fat plane between the anterior vagina and the posterior bladder wall suspicious for paravaginal tissue involvement (A: sagittal view, B: transverse view). (C, D) Colposcopy showed a papillary tumour (yellow arrows). (E) Cystoscopy showed a normal appearance of anterior wall, right lateral wall, posterior wall, right ureteric orifice and trigone. (F) The left lateral wall of the bladder demonstrated scarring from the previous TURBT with the left ureteric orifice visible in the region of scarring (yellow arrow).

**Figure 3:**
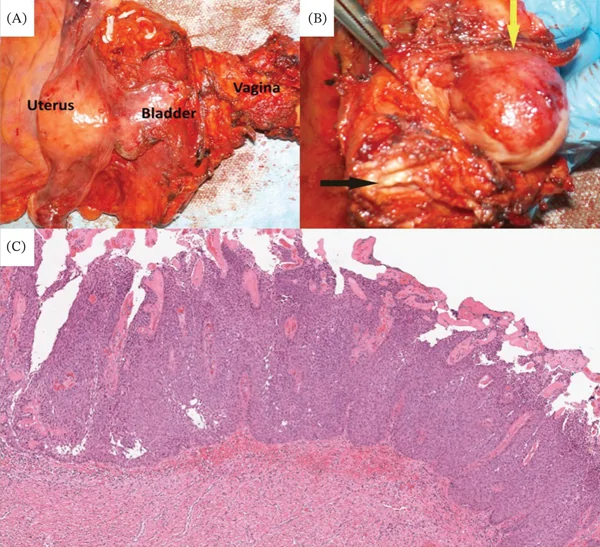
(A) Anterior exenteration specimen including the uterus, bladder and vagina. The vaginal specimen wall has been everted to show tumour at the posterior wall (yellow arrow; B) and the anterior vagina containing the urethral opening (black arrow; B). (C) H&E section of the vagina showed a superficial papillary neoplasm without evidence of mucosal invasion.

Six years later, at the age of 71, the patient presented with postmenopausal bleeding. Vaginal examination revealed an ulcerated roughened area, measuring 5 cm × 3 cm, on the left side of the vagina vault from the fornix to the introitus. A biopsy of the lesion demonstrated a papillary neoplasm morphologically similar to the original bladder UC (Figure 1). Immunohistochemical analysis was entirely supportive of UC, demonstrating 34*β*E12, CK7, CK20, GATA3, CK5 and p63 positivity, whilst p16 was negative (Figure 1). CT CAP and urography did not show any evidence of nodal or distant metastasis. An MRI scan of the pelvis showed no evidence of bladder invasion; however, was suspicious for paravaginal tissue involvement (Figure 2). Examination of the vagina on colposcopy showed papillary growth on the anterior, posterior and lateral vaginal walls, whilst cystoscopy showed no evidence of a lesion in the bladder (Figure 2). After a multidisciplinary team discussion, the patient underwent an anterior pelvic exenteration with bilateral lymph node dissection and ileal conduit formation (Figure 3). Histopathology confirmed vaginal UC with no evidence of mucosal invasion, desmoplastic stroma or inflammatory reaction (Figure 3). The bladder, despite extensive sampling of the mucosa, showed no evidence of in situ or invasive malignancy. Furthermore, there was no macroscopic or microscopic evidence of communication from the bladder or urethra. There was no evidence of lymphovascular invasion or lymph node involvement.

Whole genome SNP microarray was performed with DNA extracted from the original bladder UC and the vaginal UC to assess copy number variation and copy number neutral loss of heterozygosity (CN‐LOH). The analysis established multiple regions of variance in the bladder tumour and in the vaginal tumour and when these regions were compared across the two tumours, they were found to correspond. Both tumours demonstrated small gains of 3p, gains of 3q, CN‐LOH of distal 7q, CN‐LOH of 9p and deletion of 9p21.3 (Table 1). These findings strongly indicate clonality between the metachronous tumours.

**Table 1:** Summary of each region of chromosomal variance across both the bladder and vaginal tumours using whole genome SNP microarray.

	Variant Location	Size (kbp)	Copy Number State	Type of Variant
Bladder tumour	3p14.1	408	3	Gain
	3q11.1q29	104,335	3	Gain
	7q32.3q36.3	27,132	2	CN‐LOH
	9p24.3p13	38,979	2	CN‐LOH
	9p21.3	591	0	Deletion

Vaginal tumour	3p14.1	265	4	Gain
	3p11.1q29	107,541	3	Gain
	7q32.3q36.3	28,161	2	CN‐LOH
	9p24.3p13	38,979	2	CN‐LOH
	9p21.3	591	1	Deletion

The patient was followed up with imaging and blood work every 6 months for the first 3 years and yearly thereafter. Five years later, she has no evidence of recurrence, there are no issues with her ileal conduit and she maintains a good quality of life.

## 3. Discussion

There is a disagreement in the literature regarding the pathogenesis of vaginal UCs and whether they are secondary to bladder UCs or if they represent primary lesions. Almost all are associated with bladder UCs and frequently have a long history of multifocal bladder UCs (summarised in Table 2), indicating a likely association between the two neoplasms. Only two case reports were not associated with bladder UCs. In one case, the patient had a history of renal pelvis UC [4]. The second case had no known history of urinary tract UCs. However, the possibility of an undetected or regressed urinary tract UC cannot be fully excluded. Given the likely association between the two tumours, genetic analysis to determine clonality can be performed to establish whether non‐invasive vaginal UCs truly represent a primary lesion, or if they are secondary to bladder UCs.

**Table 2:** Summary of the case reports of vaginal UC excluding cases with associated muscle invasive UC or cases with pagetoid spread.

	Age	Vaginal UC	Synchronous Bladder UC	Previous Urinary Tract Disease	IHC	Molecular analysis	Treatment of vaginal tumour	Disease Progression
Fetissof et al., 1990 [4]	76	Non‐invasive	No	Single renal pelvis UC (non‐invasive) 4 years prior	No	No	Local resection	Vaginal recurrence
Noordzij et al., 1991 [5]	73	Non‐invasive	Yes (G1 pTa)	Multiple bladder, ureter and urethral UCs (G1‐2 pTa–pT1) over 10 years	No	No	Local resection	Vaginal recurrence
Bass et al., 1994 [6]	72	Non‐invasive	Yes (LG pTa)	Multiple bladder UCs (pTa–pT1) and renal pelvis UC (non‐invasive) over 4 years	No	No	Local resection	Vaginal Recurrence
Jendresen et al. 1995 [7]	59	Non‐invasive	No	Renal pelvis UC (pT1) and multiple bladder UCs (pTa–pT1) approximately 1 year prior	No	No	Local resection	Vaginal recurrence
Mondaini et al., 2005 [8]	82	Lamina Propria Invasion	No	Multiple bladder UCs (G2 pTa) and an upper urinary tract UC over 7 years	No	No	Not stated	No recurrence
Ogiso et al., 2006 [9]	81	Non‐invasive	Unclear	Multiple bladder UCs (pTa–pT1) over 7 years	No	No	Local resection	Vaginal recurrence
Kaneko et al., 2010 [10]	74	Non‐invasive	Yes (G2 pTa)	No	No	No	Local resection, BCG treatment and chemotherapy	Vaginal recurrence
Sigel et al., 2010 (patient 3) [11]	62	Not stated	Not stated	Multiple bladder UCs (LG, non‐invasive) over 6 years	CK7, CK20, 34*β*E12 positive	No	Not stated	Vaginal recurrence
Del Gobbo et al., 2011 [12]	51	Non‐invasive	No	No	p16 negative; p63, CK7, CK20, 34*β*E12 positive	No	Local resection	No recurrence
Alzouebi et al., 2015 [13]	80	Lamina Propria Invasion	Yes (G2 pTa)	Multiple bladder UCs (G1‐2 pTa) over 9 years	CK20, CK7, CK8/35*β*E11, 34*β*E12 positive	No	Local resection	Not stated
Warzecha et al., 2017 [14]	75	Non‐invasive	Yes (LG pTa)	No	p16 negative; GATA3, CK20 positive; patchy CK5/6 positivity	Yes	Partial vaginal resection and bilateral pelvic lymphadenectomy	Not stated
Razdan et al., 2020 [15]	92	Non‐invasive	Yes (HG pTa)	Multiple bladder, renal pelvis and ureteric UCs (LG‐HG, pTa–pT1) over 18 years	p16 patchy; p40, GATA3 positive	Unclear	Local resection and BCG treatment	Vaginal recurrence
Current Case	71	Non‐invasive	No	Single bladder UC (HG G2 pTa) 6 years prior	p16 negative; 34*β*E12, CK7, CK20, GATA3 and p63 positive	Yes	Anterior pelvic exenteration with bilateral pelvic lymphadenectomy	No recurrence

To the best of our knowledge, molecular analysis has only been performed on one other case of vaginal UC [14], in which a next generation sequencing panel was used to demonstrate corresponding point mutations of PIK3A and FGFR3 in non‐invasive bladder and vaginal UCs, demonstrating clonality. In that case, the bladder and vaginal lesions were detected synchronously; however, in our case, 6 years separated the bladder and vaginal lesions. Our case presented a unique opportunity to perform a detailed genetic analysis of both tumours, as the long interval between the presentations raised the possibility that the vaginal lesion represented a primary tumour at this site rather than a late recurrence of the bladder tumour. Additionally, we used a genome‐wide SNP microarray to assess copy number variations and regions of CN‐LOH in both metachronous tumours, which provided data on genomic evolution. All the chromosomal alterations identified were entirely comparable across the two tumours indicating shared clonality. As both tumours were non‐invasive and there was no evidence of spatial communication, this indicates that direct invasion, metastatic spread and pagetoid spread were unlikely. This suggests that tumour cells from the bladder UC have superficially ′seeded′ to the vagina prior to, or during, the original TURBT, which is consistent with the monoclonality hypothesis. These studies both indicate that vaginal UCs can spread from an original bladder UC by non‐invasive, non‐spatially connected means.

It has been postulated that tumour cells superficially seed to the vagina from the bladder via either instrumentation or urination [5, 7]. Most of the cases have a long history of bladder UCs (Table 2) prior to the development of the vaginal lesion and it has been suggested that tumour cells are implanted into the vagina during these surgical procedures. During a TURBT procedure, the vaginal introitus and lower vaginal vault are frequently and heavily exposed to bladder irrigation effluent as the bladder is repeatedly emptied and refilled. Furthermore, intraoperative bimanual examinations are standard practice; the surgeons′ gloves inevitably become saturated with this tumour‐bearing irrigation effluent, potentially transferring viable exfoliated tumour cells into the vaginal cavity. However, not all cases are associated with previous instrumentation and Warzecha et al. [14] suggest that tumour cells may seed through urine, particularly in the context of urodynamic disorders. In one case report, Ogiso et al. [9] propose that the pooling of urine secondary to labial fusion led to UC spreading over the vaginal mucosa. In the current case, as there was no history of urodynamic disorders and no visible synchronous vaginal tumour was identified at the time of the procedure, we postulate that during the initial TURBT procedure, tumour cells superficially seeded to the vagina via the retrograde escape of irrigation fluid and bimanual examination. However, we recognise that tumour cell seeding via standard physiological urine flow remains a plausible mechanism. It is also worth noting that intravesical BCG was not given following the initial TURBT because of an international supply shortage. It is possible that had this been given, this may have prevented the clonal tumour from developing. However, given the localised mechanism of action of BCG therapy, previously seeded tumour cells within the vagina may not have been affected.

The regions of variance found in the current case using whole genome SNP microarray were compared with the literature. Chromosome 9p CN‐LOH as well as deletion of 9p21.3 are commonly seen in both invasive and non‐invasive bladder UCs [17, 18]. CDKN2A (which encodes p16 and p14^ARF^) is located at 9p21.3 and homozygous deletions are found in approximately 20% of both invasive and non‐invasive bladder UCs [17]. Loss of p16 expression has been associated with increased tumour recurrence in pTa and pT1 tumours [19]. The remaining regions of variance including CN‐LOH of distal 7q and gains of 3p and 3q have not been commonly described in bladder UCs. Furthermore, the data gathered from the whole genome SNP microarray demonstrate that no further major chromosomal alterations were identified in the vaginal tumour when compared to the bladder tumour despite a 6‐year timescale between the two lesions. Although both the original bladder lesion and the subsequent vaginal lesion were of substantial size and were histologically high grade, these findings would seem to indicate a degree of molecular genetic stability, in which the vaginal tumour exhibited similar properties to the bladder tumour and did not progress to invasive disease.

An invasive vaginal UC that is secondary to invasive bladder UC commonly indicates poor prognosis, with frequent lymph node involvement and distant metastatic spread [1, 20]. What this case report highlights, in the wider context of the literature cited, is the importance of differentiating invasive and non‐invasive disease and the recognition that vaginal UCs can genuinely arise via a non‐invasive mechanism of spread. This in turn should allow for the careful consideration of more conservative management where appropriate and in the full context of multidisciplinary evidence and discussion of the patient.

In this case, whilst the possibility of non‐invasive disease was considered and a local excision was discussed, ultimately the MRI results showing a suspicion of paravaginal tissue involvement, as well as the extensive area of involvement of the vagina, meant that a local excision was clinically unfeasible. Local excision would likely have resulted in positive surgical margins and it was thought that there was a high risk of a resulting vesico‐vaginal fistula, ultimately requiring complex reconstructive procedures. A bilateral pelvic lymph node dissection was also deemed necessary to achieve full pathological staging and guide definitive oncological management. Additionally, local vaginal recurrence is also seen frequently in the literature (Table 2). Therefore, given the histological high‐grade nature of the lesion, the radiological possibility of invasive disease and extensive regional involvement, an anterior pelvic exenteration was deemed the safest oncological approach to achieve clear margins. The patient has since been followed up for 5 years and there has been no evidence of disease recurrence and she maintains a good quality of life.

In conclusion, we described a case of a non‐invasive vaginal UC occurring 6 years following treatment of a non‐invasive bladder UC. The patient has been followed up for 5 years with good oncological and functional outcomes. Molecular analysis confirmed clonality between the two lesions indicating non‐invasive tumour spread, hypothesised to occur via urination or instrumentation. This case adds further molecular genetic evidence to the literature supporting a urinary tract origin for non‐invasive vaginal UCs, rather than representing primary vaginal lesions. Furthermore, it is important to appreciate that secondary vaginal lesions can be found years after an original bladder tumour; additionally, this case demonstrated that these lesions can exhibit a degree of chromosomal stability and remain non‐invasive for years. Overall, the understanding that these non‐invasive tumours do not represent true metastatic disease is central to the prognosis and management of these patients; however, close follow up due to frequent local recurrence is required.

## Author Contributions

Cassandra Hill contributed to writing the introduction, case history and discussion, provided histopathology images and contributed to finalising the manuscript; Noman Ghazanfar contributed to writing the discussion; Andy Chuang contributed to writing the case history; Robert Gordon Hislop performed and provided expert input on the molecular genetics; Jamie Wilson provided expert input on pathological aspects and critically reviewed the manuscript; Ketan Rathod provided expert input on radiological aspects and provided informative images; Ghulam Mustafa Nandwani edited pictures, critically reviewed the manuscript and finalised the manuscript.

## Funding

This study was supported by the University of Dundee, 10.13039/100008890.

## Consent

Complete informed consent was obtained from the patient for the publication of this report and accompanying images.

## Conflicts of Interest

The authors declare no conflicts of interest.

## Supporting information


**Supporting Information** Additional supporting information can be found online in the Supporting Information section. Data S1: Detailed materials and methods used in this case are presented in the supplementary materials to maintain clarity of the main text whilst allowing full transparency and reproducibility of the findings.

## Data Availability

The data that support the findings of this study are available from the corresponding author upon reasonable request.
