# Supplementary material for: A Rare Case of a Non‐Invasive Vaginal Urothelial Carcinoma Demonstrating a Clonal Relationship With a Metachronous Non‐Invasive Bladder Urothelial Carcinoma
Source: Case Rep Pathol. 2026 Sep 17;2026:4360431. doi: 10.1155/crip/4360431 (PMC13584327; doi:10.1155/crip/4360431)
Supplement: Supplementary file 1 [file CRIP-2026-4360431-s001.docx]

# Supplementary Material

## Materials and Methods

Immunohistochemistry was performed on formalin fixed paraffin embedded (FFPE) tissue blocks from the vaginal biopsy. The following immunohistochemical markers were used: CK7 (Roche; clone SP52), CK20 (Agilent Dako; clone KS20.8), GATA3 (Roche; clone L50-823), 34βE12 (Agilent Dako; clone 34β12), p16 (Roche; clone CINtec p16), p63 (Leica; clone p63), CK5 (Agilent Dako; clone D5/16B4).

FFPE tissue blocks with the highest tumour cell content were identified from both the original bladder tumour and the vaginal resection specimen. DNA was extracted and purified from the FFPE tissue using the Maxwell® DNA FFPE Kit (Promega). This was performed following the manufacturers protocol (Promega, 2017). Whole genome SNP microarrays were performed on DNA extracted from both the vaginal and bladder tumours using the FFPE OncoScan^TM^ Assay (Thermo Fischer Scientific) following the manufacturers manual (Thermo Fischer Scientific, 2015). A 10Mb cut off was used to determine regions of significant LOH. The ChAS software (Thermo Fischer Scientific) was used to visualise and interpret the results of the assay, demonstrating copy number and heterozygosity by calculating log2 copy ratios and B Allele Frequency respectively.
